# Cardio-renal effect of dapagliflozin and dapagliflozin- saxagliptin combination on CD34 + ve hematopoietic stem cells (HSCs) and podocyte specific markers in type 2 diabetes (T2DM) subjects: a randomized trial

**DOI:** 10.1186/s13287-025-04130-x

**Published:** 2025-01-26

**Authors:** Seshagiri Rao Nandula, Arad Jain, Sabyasachi Sen

**Affiliations:** 1 Department of Medicine, Veterans Affairs Medical Center, Washington, DC USA; 2 Department of Medicine, George Washington University, Washington, DC USA; 3 Department of Biochemistry, George Washington University, Washington, DC USA

## Abstract

**Introduction:**

Effects of Dapagliflozin (Dapa) and Dapagliflozin-Saxagliptin combination (Combo) was examined on peripheral blood derived CD34 + Hematopoetic Stem Cells (HSCs) as a cellular CVD biomarker. Both Dapa (a sodium-glucose co-transporter 2 or SGLT2, receptor inhibitor) and Saxagliptin (a Di-peptydl-peptidase-4 or DPP4 enzyme inhibitor) are commonly used type 2 diabetes mellitus or T2DM medications, however the benefit of using the combination has not been evaluated for cardio-renal risk assessment, in a real-life practice setting, compared to a placebo.

**Hypothesis:**

We hypothesized that Dapa will improve the outcomes when compared to placebo and the Combo maybe even more beneficial.

**Methods:**

This is a pilot study evaluating low dose Dapagliflozin 10 mg or low dose Dapa + low dose Saxagliptin combination. 15 subjects were enrolled in 16 weeks, double-blind, three-arm, randomized placebo matched trial, with 10mg **Dapa** + Saxa placebo (*n* = 4), 10 mg Dapa + 5 mg Saxa (*n* = 5) **Combo**, And Dapa placebo + Saxa placebo (*n* = 6), **Placebo** groups. T2DM subjects (age 30–70 yrs) with HbA1c of 7–10%, were included. CD34 + HSC number, migration, mRNA expression along with biochemistry and urine exosomes were measured. Data were collected at week 0, 8, and 16. For statistics, a mixed model regression analysis was used.

**Results:**

Significant HbA1c (*p* = 0.0357) reduction was noted in Combo group versus Dapa alone and Placebo. hsCRP levels (*P* = 0.0317) and IL-6, two important inflammatory molecules, were significantly reduced in both Dapa and Combo vs. Placebo. Leptin levels decreased significantly in both Dapa alone (*p* = 0.035) and Combo group(*p* = 0.015), vs. Placebo, however the Adiponectin levels were higher in Dapa alone group. Dapagliflozin alone reduced lipid parameters significantly particularly triglyceride (TG) when compared to placebo, with resultant visit 3 values at 99.5 *±* 7.2 vs. 129 *±* 12.3 and LDL/HDL ratio values were similar at 2.18 *±* 0.08 vs. 2.13 *±* 0.15. CD34 + cell migration improved significantly in both Dapa alone (*p* = 0.05) and Combo group (*p* = 0.05) vs. Placebo.

**Conclusions:**

Several parameters showed significant improvement with both Dapa alone and Combo compared to placebo. However, when all outcome measures were taken into account, other than glycemic control the Combo didn’t seem to offer any further benefit, over Dapa alone. Therefore, contrary to our initial hypothesis we do not believe the more expensive Dapa + Saxa combination offers any specific cardiovascular benefit compared to Dapagliflozin alone. However it is noteworthy that both Dapa and its combination with Saxagliptin showed significant improvement compared to placebo in T2DM, particularly when progenitor cell based numbers and function were analyzed and taken into account.

**Trial Registration:**

The trial was registered with Clinical Trials.gov number NCT03660683, last updated 06052023.

## Background

According to the National Diabetes Statistics Report [1], more than 34 million people in the United States have been diagnosed with type 2 diabetes. Risk factors for type 2 diabetes mellitus (T2DM) include excess body weight and physical inactivity. Current projections estimate that nearly 700 million adults will be living with diabetes in 2045, reaching proportions that resemble a world epidemic [2]. Diabetes is closely associated with oxidative stress, inflammation, and as a result endothelial dysfunction and cardiovascular complications such as heart failure and kidney disease [3]. Both T2DM and chronic kidney disease (CKD) are conditions that are responsible for vascular damage and vascular complications and leads to pro-thrombotic states. As we develop therapeutic options for glycemic control and diabetes related complications such as chronic kidney disease (CKD), it is important to establish whether these treatments are also addressing cardiac-kidney-metabolic (CKM) components rather than metabolic or glycemic control alone in patients with diabetes. In this clinical trial we have specifically focused on endothelial progenitor cells (EPCs), defined as peripheral blood derived hematopoetic progenitor cells that are CD34 + ve, as a CKM health-indicator along with usual plasma based bio-markers used as described in standard of diabetes care guidelines [3–10].

Previous studies by our laboratory [9, 10] have investigated Endothelial Progenitor Cells (EPCs), defined as CD34 + ve hematopoietic stem cells (HSCs), as a marker for cardiovascular disease (CVD). Studies have shown that circulating CD34 + EPC number, function and mRNA expression can act as a reliable and robust cellular biomarker compared to serum-based endothelium specific biomarkers for monitoring endothelial dysfunction in type 2 diabetes (such hs-C-reactive protein (hs-CRP) and interleukins). This is `particularly due to the slow or delayed response to changes in plasma based cardiometabolic disease (CVD) markers commonly used in clinical practice [4–8]. EPCs or CD34 + HSCs are specialized stem/progenitor cells [6] responsible for repair of the endothelial cell lining of blood vessels and angiogenesis. These cells can be harvested from peripheral whole blood derived mononuclear cells (MNC) by positive sorting for CD34 peripheral cell surface receptor [9–13]. We and others have shown that high glucose environment, as seen in diabetes mellitus (either type 1 or 2), leads to functional impairment of circulating EPCs by activating or upregulating p53 gene (a prominent gene associated with apoptosis) and it’s downstream cascade of apoptosis [13]. Our lab has focused on studying changes in EPC populations in T2DM [10–12] with or without various classes of diabetes medications and non-medication interventions such as exercise.

For T2DM treatment, metformin is usually used as the first-line agent to control hyperglycemia in patients. Sodium-Glucose Co-transporter 2 Inhibitors (SGLT2is) are one class of medication that has demonstrated ability to improve glycemic control, obesity and hypertension as reported by `several research groups, including ours [8, 14–17]. Previous studies from our laboratory have shown that low dose Canagliflozin (an SGLT2i) has a beneficial effect on EPC function, serum biochemistry, and urinary podocyte specific exosomes in type 2 diabetes [9]. This demonstrates the potential for SGLT2i as a treatment not only for treatment of diabetes associated hyperglycemia, but also to improve related renal and cardiovascular complications as well.

Dipeptidyl peptidase-4 (DPP-4) enzyme inhibitors, are a class of oral anti-diabetic medications, that have been shown to achieve improved glycemic control by lowering HbA1C, without causing hypoglycemia. DPP-4 enzyme degrades incretins such as GLP1 and GIP, including chemotactic factors such as SDF-1ɑ (stromal derived factor). Therefore, use of DPP-4 inhibitor is expected to be associated with increased bioavailability of SD-1ɑ. This may help “homing-in” of EPCs to the damaged endothelial sites. Recent studies by our lab have shown that treatment with Linagliptin (a DPP-4 inhibitor) for 12 weeks promote an increase in CXCR4 expression in EPCs with improvement in vascular and renal parameters for patients with diabetic kidney disease [10].

Therefore we postulated that combination of a similar SGLT2i such as Dapagliflozin in combination with DPP4i such as saxagliptin may have added cardio-metabolic beneficial effect. We therefore conducted a pilot 16-week, three-armed, double-blind randomized controlled trial to evaluate whether a combination of 10 mg Dapagliflozin (SGLT2i) treatment with 5 mg Saxagliptin (DPP-4 inhibitor) treatment would improve EPC number and function and also improve usual standard of care biochemical analysis, when compared to Dapagliflozin treatment alone or placebo treatment.

## Methods

### Trial design and oversight

**Fig. 1:**
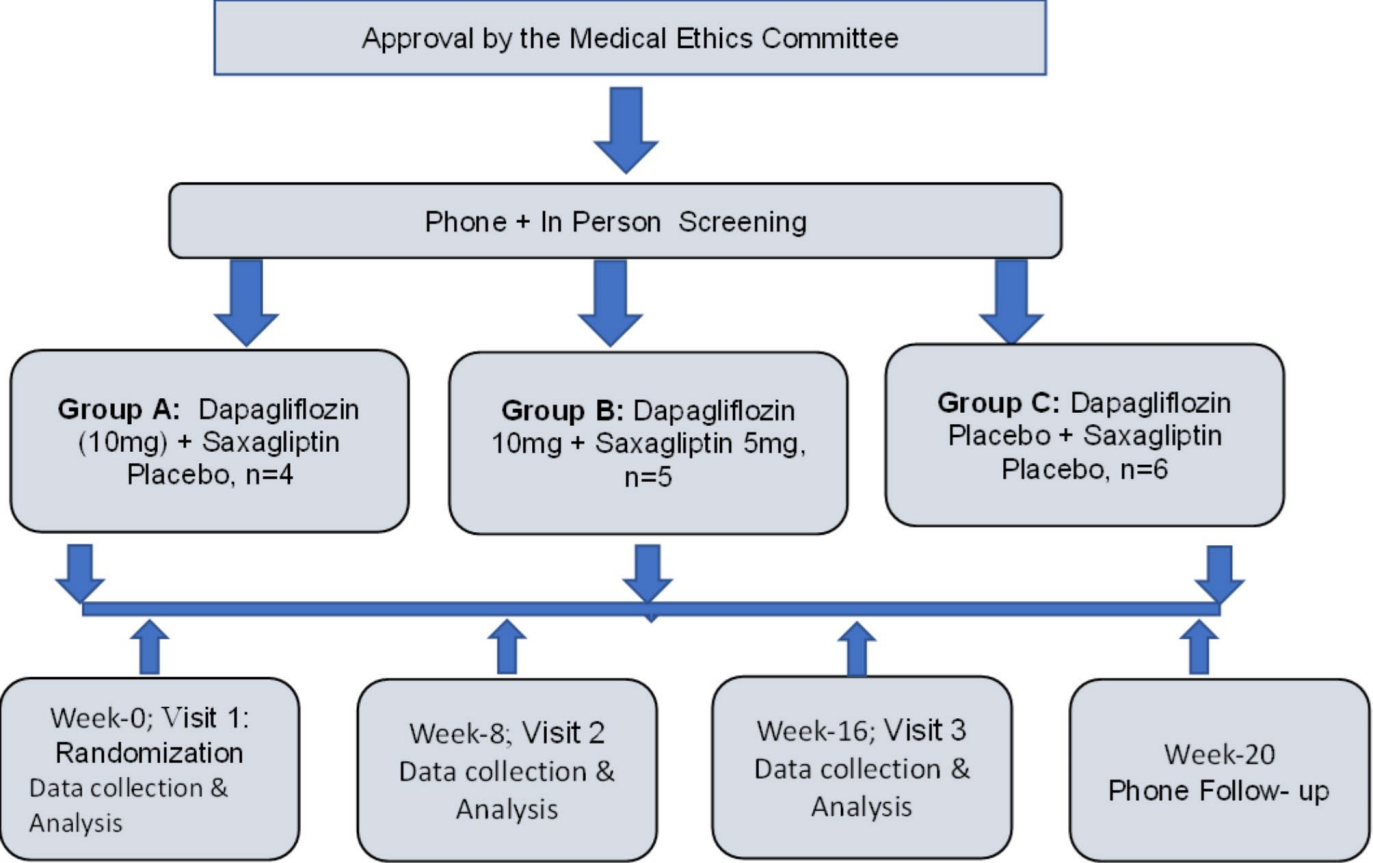
Trial Design. Three groups of medication with 20-week design

This is a phase 4 pilot clinical study, with three arm, single site, parallel group, double blind, placebo controlled randomized clinical trial comparing low combination doses of Dapagliflozin 10 mg and Saxagliptin tablets, taken orally, once daily, with matching control groups of Dapagliflozin 10 mg with Saxagliptin placebo, or matching placebo for both medications. We specifically choose low doses of both agents so that safety of our subjects is ensured. See Fig. 1 for study design.

### Ethics approval

The study was conducted in accordance with Good Clinical Practice guidelines set forth by the International Conference of harmonization and any local regulatory guidelines with the approval and oversight of the George Washington University Institutional Review Board. The study was approved by the George Washington University Institutional Review Board. The trial was funded by AstraZeneca, LLC and conducted by the Investigator-Sponsor Dr. Sabyasachi Sen at the George Washington University.

Subjects were initially pre-screened to assess eligibility. Once preliminary eligibility was determined, they were brought in for a screening visit to confirm eligibility via interview, medical record check and laboratory workup once the subject signed the informed consent. The subjects (*n* = 15 in total) were then enrolled into one of three arms of the study: 10 mg Dapagliflozin, 10 mg Dapagliflozin and 5 mg Saxagliptin combination, or matching placebo.

### Enrollment numbers

This was a small pilot study. 4 subjects were enrolled into the active dapagliflozin group, 5 subjects enrolled in the dapagliflozin and saxagliptin combination group, and 6 subjects were enrolled into the placebo group.

There were 3 study visits in total, first at week 0, second at week 8 (mid-point) and third and last visit at week 16. All three of the visits had same assessments. The assessments that were done were: vital measurements, adverse event (AE) check and a peripheral blood draw. Approximately 60 ml of blood was drawn for CD34 + ve endothelial progenitor cell harvesting and routine blood work.

Other parameters tested were resting metabolic rate (RMR, energy expenditure), measurement of waist to hip ratio, urine sample collection, Tanita body composition scale, pulse wave analysis and pulse wave velocity to determine arterial stiffness. Subjects were advised to adhere to 150 min of weekly aerobic exercise and their activity levels were monitored using ACTi graph activity monitor.

A follow up phone call visit was done 30 days from the last in-person visit to assess for any residual adverse events (AE).

### Participants

Subjects were included if they were between 30 and 70 years old, with a diagnosis of Type 2 Diabetes Mellitus (T2DM). HbA1c inclusions were between 7.0 and 10.0%. Their baseline medications were stable dose of Insulin (either short acting or long acting) and/or Metformin (1–2 g/day). A stable dose was considered to be at least the maximum labeled dose or a dose that is not associated with unacceptable side effects. Patients with BMI between 25 and 39.9 kg/m2 were included, thereby excluding severe obesity. Only patients with impaired renal function were included, that is Chronic Kidney Disease (CKD) stages 1 to 3, with lowest eGFR cut-off of 30 ml/min/1.73 (GFR, as calculated by MDRD formula). During the duration of the study alterations in baseline medications such as insulin dose adjustments were done, to keep HbA1C between 7 and 8% across all subjects over the 16 week period of medication intervention.

Any patients with Type I diabetes, history of Diabetic ketoacidosis, low hematocrit (less than 28 units), history of recent pancreatitis or cancer, recent coronary or cerebrovascular event within last 6 months, use of consistent high dose steroid medications, or untreated thyroid disease were excluded.

Additional Inclusion and Exclusion criteria can be found in Appendix.

### Outcome objectives and methods

Several of the outcome measures described here are similar to our previous clinical studies on T2DM [9–11].

The primary objective was to ascertain whether 16 weeks of Dapagliflozin treatment, with or without Saxagliptin combination, improves CD34 + ve cell number, (CD34 + ve number, %CD34 + ve of total Mononuclear Cell population) function (cell migration function in response to SDF1α) and gene expression. These cellular outcome measures were correlated to improvements in 24 h urinary protein estimation and serum Creatinine Clearance and other serum markers of vascular health such as Serum Biochemistry (CMP, IL6, hsCRP, Leptin, adiponectin, Serum insulin, TNFα), and measures of glycemic control .

The secondary objective was to correlate the cellular outcome measures with other non serum measures of endothelial function such as Arterial Stiffness [measured by pulse wave analysis (Augmentation Index) and pulse wave velocity (m/s)]. Adiposity (as % body fat), resting energy expenditure (in kcal) were measured using Tanita scale and Reevue machine, respectively [9–11].

### Body composition measurement

Body composition was measuring using Tanita™ BF-350 Body Composition Scale and manually. Manual measurement included height, waist circumference, hip circumference. Tanita scale (Tanita Corporation of America, Inc, USA) uses a bio-impedance electrical impulse to measure body fat percent, fat mass (kg), fat free mass (kg), percent body water, water mass (kg) alongside weight. It then calculates the BMI and estimated basal metabolic rate [9–11].

### Basal metabolic rate measurement

Resting Emergency Expenditure (REE) was measured using KORR REEVUE (Korr Medical Technologies, USA). The test was conducted with the subject sitting upright and well rested. The subject was instructed to keep a tight seal around the mouthpiece and use the nose clip to avoid breathing in from the nose. The test took approximately 10 min. It calculated estimated REE, predicted REE, estimated Total Energy Expenditure (TEE), VO2 Max. Tidal Volume, Breathing rate, and estimated calorie intake per day [9–11].

### Arterial stiffness

This parameter was measured using Atcor Sphygmocor CP system (Atcor Technologies, USA). We obtained two outcome measurements: pulse wave velocity (PWV) and pulse wave analysis (PWA). The patient was supine on the examination table during these assessments [9–11].

Pulse wave analysis (PWA) was measured on the left Radial Artery with the subject at supine position. At least three readings were taken with Operator Index ≥ 80. Measurement includes Augmentation Index (AI), Augmentation Index adjusted for Heart Rate of 75 (AI-75), Augmentation Pressure (AP), Aortic and Radial reading of systolic, diastolic, pulse pressure and mean pressure [9–11].

Pulse wave velocity (PWV) was measured with the subject supine. This measurement requires a distal and proximal artery. Distal was used as right femoral artery with proximal being the left carotid. Index and ring fingers were used to manually localize the pulse, sometimes an arterial Doppler was used to localize the femoral pulse on patients with challenging body habitus. Once a stable pulse waveform was observed, the probe position was kept stable for 20 more pulses before the reading was finalized. Measurements from the sternal/aortic notch to the distal and proximal pulse locations were taken, while the software measured the delay between the two pulses. Three readings were taken with standard deviation of less than 10%. The result reported a velocity in m/s, alongside the standard deviation [9–11].

### Biological sample and vital collection

A venous blood sample was collected from the Antecubital fossa. About 80 ml of blood was collected. 60 ml for EPC analysis and 20 ml for standard of care blood works which included Basic Metabolic Panel, Lipid Panel, HbA1c, hsCRP, IL6, Adiponectin and Insulin. Enzyme linked immune assay (ELISA) was performed to analyze serum GLP1 and SDF1α using ELISA Immunoassay kit (Raybiotech, Norcross, GA) for GLP1 and Sandwich ELISA (EHCXCL12A, Thermo Scientific, USA) for SDF1α. Urine sample was collected for urine Microalbumin and Creatinine ratio. Vitals were gathered on the left arm, Systolic Pressure, Diastolic Pressure and heart Rate, along with sublingual temperature. Serum nicotinamide adenine dinucleotide (NAD/NADH) were measured by using NAD/NADH assay kit from Abcam, MA, USA (Catalog No. ab65348) and Ketone bodies were from serum were measured by using Ketone Body Assay Kit from Millipore Sigma, MO, USA (Catalog No. MAK134).

### Polyethylene glycol (PEG) enrichment of extracellular vesicles

The cells debris and large apoptotic bodies were removed from the urine samples by centrifugation at 500×g for 5 min followed by 3000×g for 30 min at 4 °C. Transfer supernatant into ultracentrifugation tubes and centrifuged at 100,000×g at 4 °C for 75 min (Optimal XPN-100 centrifuge, Beckmann Coulter Inc, USA). After ultracentrifugation the pellet was dissolved in RIPA buffer with protease inhibitor cocktail and stored the sample at − 80 °C for further analysis.

### Extracellular vesicle characterization and western blotting

Extracellular vesicles were isolated by ultracentrifugation and identified by the expression of CD9, CD81, CD63 AND HSP70 markers in western blot. Extracellular vesicle extracts were fractionated by SDS-PAGE and transferred to a polyvinylidene difluoride membrane using a transfer apparatus according to the manufacturer’s protocols (Bio-Rad, USA). After incubation with 5% nonfat milk in TBST (Bio-Rad, USA) (10 mM Tris, pH 8.0, 150 mM NaCl, 0.5% Tween 20) for 60 min. The membrane was washed once with TBST and incubated with antibodies against CD9 (1:1000), CD81 (1:1000), CD63 (1:1000), HSP70 (1:1000) (Sysetem Biosciences, USA), anti-podocalyxin (PODXL, 1:1000), anti-wilms tumor protein (1:1000) and anti-Nephrin antibody (1:1000) at 4 °C for 12 h (Abcam, USA). Membranes were washed three times for 10 min and incubated with a 1:20,000 dilution of horseradish peroxidase-conjugated goat anti-rabbit antibody for 90 min at room temperature. Blots were washed with TBST three times and developed with Pierce ECL kit (ThemoFisher Scientific, USA) [9, 10].

### Cellular and clinical assessments

#### CD34 + ve endothelial progenitor cell analysis

Peripheral blood samples (approximately 60 ml) were drawn from patients and phosphate buffered saline (1:1) was added. Identification and quantification of circulating cell phenotypes was performed on fresh blood samples, within 3 h after collection, using flow cytometry. Briefly, mononuclear cells (MNCs) were then isolated from whole blood using a Ficoll density centrifuge method. MNCs were counted and aliquot was used for CFU-Hill colony formation assay following the manufacturer’s instruction (Stem Cell Technologies, Vancouver, BC, Canada). Colony forming units were counted at day 14. A fraction of the MNC were stained with fluorescein isothiocyanate (FITC)-conjugated antihuman CD34, Allophycocyanin (APC) conjugated antihuman CD184 (CXCR4) and FITC conjugated antihumanCD31 antibodies (Miltenyi Biotec GmbH, Bergisch-Gladback, Germany) in order to analyze specific progenitor cell surface marker (CD34) and mature endothelial cell surface markers (CD31) or receptor for SDF1a ligand, CXCR4) by flow cytometry. After gating mononuclear cells in the side scatter (SSC)-A vs. forward scatter (FSC)-A plot, CD34/CD184 single- and double-positive cells were identified. Cells were acquired on a fluorescence-activated cell sorter (FACS) Canto instrument (Becton Dickinson, USA) and scored with the FloJo software [9, 10].

To isolate EPCs (CD34 + ve), MNCs were magnetically sorted through a column after cells were stained with CD34 + ve microbeads antibody (Miltenyi Biotec GmbH, Bergisch Gladback, Germany). An aliquot of CD34 + ve cells were then stained with trypan blue and counted using an Auto Cellometer Mini (Nexcelom Bioscience, USA) to assess viability [9–11].

CD34 + ve gene expression analysis was performed by quantitative reverse transcriptase polymerase chain reaction (qRT-PCR) as previously described. CD34 + ve cell total mRNA was extracted and purified using the RNeasy Minikit (Qiagen, Germany). mRNA was then converted into cDNA by using the high capacity cDNA reverse transcriptase kit (Thermo Fisher Scientific, MA) Possible gene expression changes promoted by the pharmacological intervention were assessed by a CFX96 real-time PCR system (Bio-Rad, CA) using Taqman Universal Master Mix II (Thermo Fisher Scientific, USA) and inventoried probes. The gene expression analysis included genes associated with antioxidants, apoptosis, endothelial functions, chemotaxis, inflammation and endothelial lineage or endothelial function related cell surface markers. The expression of each individual gene was normalized to either housekeeping 18 S or GAPDH and calculated using C-ddct method considering the difference in cycle threshold between visit 2 or 3 and baseline (visit 1).

The migratory capacity of CD34 + ve was evaluated using the CytoSelect 24-well Cell Migration Assay kit (Cell Biolads, Inc., San Diego, CA). Cells were suspended in Serum free media and seeded at 100,000 cells per insert. Migration of the cells through a 3 μm polycarbonate membrane to the wells containing a serum-free media (control) and chemoattractant SDF-1α (10 or 100 ng/ml) (from Sigma-Aldrich, USA) was assessed after cells were kept overnight in incubator. Migratory cells were dissociated from the membrane and subsequently lysed and quantified by fluorescence (480 nm/530 nm) using CyQuant GR dye (Cells Biolabs, Inc, USA). The fluorescence ratios between cells exposed to the chemotactic factor and cells exposed to chemoattractant-free media (control) along the visits were used to analyze the migratory capacity of the cells [9–11].

We also estimated colony forming units (CFU) from MNC population [18, 19].

Details on clinical and cellular outcome measures are categorized in Tables 1 and 2 respectively.

**Table 1:** Clinical parameters

**Placebo**							
	**Visit 1**	**SEM V1**			**Visit 3**	**SEM V3**	***P*** **value(V1/V3)**
Systolic	136.83	3.01			132.00	1.17	0.29
Diastolic	82.50	2.23			80.40	1.50	0.39
Pulse	80.00	1.58			87.40	1.00	0.08
Temp	98.45	0.06			98.50	0.07	0.42
BMI	35.32	0.82			37.44	1.21	0.29
Weigh Kgs	99.32	3.03			106.45	3.87	0.29
Waist in CM	108.80	2.04			117.33	2.52	0.21
Hip in CM	117.20	2.03			131.00	2.83	0.13
Avg PWV	8.68	0.34			9.55	0.22	0.28
Avg Aug Index_75	26.42	2.09			27.75	1.00	0.45
Avg Aug Index	26.92	2.32			25.65	0.15	0.45
Avg Aug Pressure	14.44	2.07			6.77	0.77	0.18
Glucose	157.40	4.81			184.80	7.75	0.15
Uric Acid	5.00	0.22			5.72	0.16	0.17
BUN	10.00	0.54			13.00	0.70	0.11
Creatinine (Serum)	0.75	0.02			0.81	0.03	0.26
EGFR	106.50	3.86			102.60	5.23	0.41
Sodium	139.17	0.32			137.00	0.33	0.05
Potassium	4.28	0.03			4.22	0.07	0.39
Chloride	101.67	0.53			100.40	0.53	0.27
AST (SGOT)	31.33	2.95			35.40	4.13	0.38
ALT (SGPT)	42.33	4.69			39.80	4.33	0.44
Cholesterol	150.00	2.14			149.40	5.63	0.48
Triglycerides	137.83	10.21			128.80	12.33	0.41
HDL Cholesterol	45.83	1.74			40.80	1.36	0.20
VLDL Cholesterol	27.00	2.04			25.40	2.52	0.43
LDL Cholesterol	77.17	2.60			83.20	3.95	0.31
LDL/HDL	1.81	0.11			2.13	0.15	0.26
CRP	12.21	3.77			4.26	0.35	0.26
IL6	3.42	0.19			5.14	0.47	0.10
HbA1c	8.57	0.27			8.80	0.27	0.41
Leptin	66.37	7.56			80.96	10.35	0.33
Adiponectin	5.88	0.95			5.02	0.79	0.40
Insulin	19.90	3.23			20.16	2.74	0.49
TNFa	1.45	0.09			1.75	0.18	0.28
**Dapagliflozin**							
	**MEAN V1**			**SEM V1**	**MEAN V3**	**SEM V3**	***P*** **value(V1/V3)**
Systolic	104.00			1.41	123.67	5.11	0.15
Diastolic	69.50			0.18	75.00	1.75	0.19
Pulse	86.50			1.59	73.67	2.02	0.08
Temp	98.30			0.00	98.17	0.07	0.29
BMI	29.40			1.32	29.23	1.38	0.49
Weigh Kgs	96.39			5.28	95.80	5.61	0.49
Waist in CM	109.67			5.34	106.00	4.99	0.42
Hip in CM	109.33			2.67	111.33	3.64	0.43
Avg PWV	8.45			0.37	8.23	0.16	0.41
Avg Aug Index_75	11.17			1.21	12.00	1.00	0.41
Avg Aug Index	11.00			0.66	13.67	2.13	0.32
Avg Aug Pressure	2.67			0.14	4.00	0.66	0.22
Glucose	152.33			20.74	113.50	4.77	0.29
Uric Acid	4.90			0.20	4.15	0.16	0.18
BUN	19.00			0.75	18.00	0.71	0.37
Creatinine (Serum)	1.05			0.06	0.93	0.01	0.28
EGFR	87.67			5.68	96.50	4.42	0.34
Sodium	138.67			1.18	141.50	0.88	0.26
Potassium	4.07			0.04	4.15	0.02	0.27
Chloride	103.67			1.13	103.50	0.53	0.48
AST (SGOT)	15.33			0.80	18.00	0.00	0.17
ALT (SGPT)	16.33			0.76	17.50	0.18	0.32
Cholesterol	150.00			2.14	149.40	5.63	0.48
Triglycerides	137.83			10.21	128.80	12.33	0.23
HDL Cholesterol	45.83			1.74	40.80	1.36	0.29
VLDL Cholesterol	27.00			2.04	25.40	2.52	0.23
LDL Cholesterol	77.17			2.60	83.20	3.95	0.38
LDL/HDL	1.81			0.11	2.13	0.15	0.30
CRP	12.21			3.77	4.26	0.35	0.15
IL6	3.42			0.19	5.14	0.47	
HbA1c	8.57			0.27	8.80	0.27	0.47
Leptin	66.37			7.56	80.96	10.35	0.28
Adiponectin	5.88			0.95	5.02	0.79	0.30
Insulin	19.90			3.23	20.16	2.74	0.32
TNFa	1.45			0.09	1.75	0.18	0.48
**Combination**							
	**MEAN V1**		**SEM V1**		**MEAN V3**	**SEM V3**	***P*** **value(V1/V3)**
Systolic	125.00		1.98		132.00	4.94	0.31
Diastolic	78.00		0.49		73.75	1.53	0.16
Pulse	75.00		2.20		71.00	1.93	0.30
Temp	97.93		0.05		98.05	0.01	0.17
BMI	33.65		1.06		34.30	1.31	0.44
Weigh Kgs	101.00		3.62		99.10	3.29	0.44
Waist in CM	115.33		3.52		115.67	2.89	0.49
Hip in CM	110.67		1.68		111.33	1.47	0.46
Avg PWV	9.22		0.25		8.10	0.14	0.17
Avg Aug Index_75	21.00		1.40		30.00	3.96	0.21
Avg Aug Index	20.90		1.22		34.00	2.83	0.08
Avg Aug Pressure	7.88		0.78		13.50	1.56	0.14
Glucose	147.40		4.11		106.40	5.43	0.01
Uric Acid	5.22		0.36		4.50	0.21	0.23
BUN	18.60		2.10		17.80	1.83	0.45
Creatinine (Serum)	1.04		0.09		1.07	0.06	0.45
EGFR	84.60		6.25		78.00	4.88	0.36
Sodium	141.20		0.52		139.40	0.30	0.11
Potassium	4.12		0.04		4.14	0.02	0.43
Chloride	103.40		0.69		286.80	82.20	0.17
AST (SGOT)	26.80		2.04		25.20	1.31	0.39
ALT (SGPT)	41.40		3.87		31.80	1.87	0.17
Cholesterol	129.00		5.67		119.80	3.11	0.27
Triglycerides	179.80		33.90		188.20	33.53	0.47
HDL Cholesterol	38.20		1.81		37.80	2.52	0.48
VLDL Cholesterol	20.25		0.93		30.80	4.38	0.19
LDL Cholesterol	57.75		4.76		51.20	3.12	0.32
LDL/HDL	17.50		3.20		13.22	0.96	0.49
CRP	1.43		0.31		1.41	0.25	0.49
IL6	4.36		0.62		2.43	0.40	0.19
HbA1c	7.24		0.10		6.64	0.08	0.03
Leptin	30.78		3.70		25.60	3.50	0.33
Adiponectin	2.02		0.22		2.10	0.26	0.46
Insulin	18.24		2.79		15.76	1.96	0.38
TNFa	1.46		0.10		1.45	0.09	0.49

**Table 2:** Cellular

**Placebo**									
	**Visit 1**		**SEM V1**	**Visit 3**		**SEM V3**		***P*** **value(V1,V3)**	
**Migration assay**	0.54		0.00	0.25		0.07		0.14	
**CFU**	9.70		1.28	4.13		0.45		0.11	
**CAT**	1.10		0.51	1.86		1.74		0.22	
**CXCL12**	1.26		1.01	5.23		8.52		0.23	
**CXCR4**	1.78		1.84	1.61		1.85		0.45	
**EDN1**	1.11		0.50	1.01		0.22		0.41	
**GPX3**	2.20		2.51	4.36		6.00		0.27	
**IL6**	1.27		0.97	1.42		1.42		0.44	
**KDR**	1.49		1.59	1.34		1.25		0.46	
**NOS3**	1.03		0.26	1.06		0.49		0.46	
**P21**	1.12		0.69	1.26		1.13		0.44	
**PECAM1**	2.18		2.98	1.56		1.38		0.36	
**SOD2**	1.04		0.37	1.71		2.26		0.29	
**TNF**	2.96		4.41	2.20		3.08		0.40	
**TP53**	1.17		0.70	2.00		1.93		0.26	
**VEGFA**	2.47		3.88	1.96		2.20		0.42	
**CD34-FITC**	3.55		0.29	1.74		0.31		0.16	
**CD184-APC**	73.40			68.37		3.44			
**CD31-FITC**	63.70		6.06	60.68		6.86		0.47	
**CD34+CD184+**	2.38		0.49	20.11		6.05		0.28	
**CD31+CD184+**	66.10			63.28		2.85			
**Nephrin/CD9**	0.55		0.11	0.67		0.13		0.43	
**PODXL/CD9**	3.13		0.70	0.80		0.01		0.26	
**Dapagliflozin**									
	**Visit 1**		**SEM V1**		**Visit 3**		**SEM V3**		***P*** **value(V1, V3)**
**Migration assay**	0.53		0.05		0.68		0.03		0.12
**CFU**	18.88		6.71		5.33		1.09		0.22
**CAT**	1.43		1.28		1.42		1.20		0.50
**CXCL12**	1.01		0.22		15.95		26.74		0.19
**CXCR4**	1.13		0.56		1.31		0.90		0.37
**EDN1**	1.14		0.65		1.93		2.00		0.24
**GPX3**	1.13		0.70		1.52		1.25		0.31
**IL6**	1.22		0.88		1.01		0.16		0.35
**KDR**	1.18		0.62		1.89		1.86		0.25
**NOS3**	1.12		0.59		1.72		2.13		0.30
**P21**	1.24		0.97		1.48		1.14		0.38
**PECAM1**	1.36		0.97		1.35		0.99		0.49
**SOD2**	1.05		0.42		1.07		0.41		0.47
**TNF**	1.85		1.27		1.19		0.71		0.20
**TP53**	1.47		1.46		1.59		1.27		0.45
**VEGFA**	1.29		1.09		1.31		0.97		0.49
**CD34-FITC**	0.61		0.19		1.15		0.33		0.34
**CD184-APC**	63.85		7.73		58.40		7.78		0.44
**CD31-FITC**	87.35		3.24		69.00		10.29		0.30
**CD34+CD184+**	0.65		0.20		1.00		0.30		0.38
**CD31+CD184+**	55.30		3.71		56.00		10.64		0.49
**Nephrin/CD9**	1.64		0.04		1.16		0.10		0.12
**PODXL/CD9**	3.30		0.22		2.59		0.43		0.33
**Combination**									
	**Visit 1**		**SEM V1**		**Visit 3**		**SEM V3**		***P*** **value(V1, V3)**
**Migration assay**		0.38	0.10		0.75		0.02		0.13
**CFU**		12.63	1.39		9.70		2.32		0.34
**CAT**		1.12	0.56		1.72		1.92		0.29
**CXCL12**		1.02	0.25		1.01		0.17		0.49
**CXCR4**		1.52	1.42		1.08		0.48		0.32
**EDN1**		1.08	0.58		1.00		0.01		0.40
**GPX3**		1.22	0.77		1.65		1.87		0.36
**IL6**		1.91	1.62		1.03		0.32		0.20
**KDR**		1.54	1.66		1.04		0.40		0.36
**NOS3**		1.19	0.87		1.20		0.86		0.49
**P21**		1.28	1.02		1.38		1.30		0.45
**PECAM1**		1.50	1.63		1.21		0.79		0.38
**SOD2**		1.23	0.93		1.24		0.98		0.49
**TNF**		1.48	1.52		1.10		0.58		0.35
**TP53**		1.47	1.38		1.31		1.03		0.44
**VEGFA**		5.34	9.53		1.22		0.74		0.25
**CD34-FITC**		2.35	0.36		3.17		0.28		0.22
**CD184-APC**		54.78	6.18		62.78		4.85		0.34
**CD31-FITC**		63.34	6.11		81.94		5.49		0.17
**CD34+CD184+**		1.92	0.29		3.08		0.15		0.08
**CD31+CD184+**		39.10	5.83		51.62		5.64		0.27
**Nephrin/CD9**		0.66			0.49		0.10		
**PODXL/CD9**		2.77			2.10		0.48		

### Statistical analysis

Continuous variable distributions were examined for skewness or outliers using histograms. When these were present, we log-transformed the variables. Outliers > 5SD from the mean were capped at 5SD from the mean. We used 2-tailed between-groups t-tests to examine differences between treatment groups at baseline on continuous variables, and either chi-square or the Fishers Exact test for categorical variables. To examine differences between treatment groups across all time points, as well as time effects, and whether the slope of change over time differed between treatments, we used random effects mixed model regression, examining the main effects of treatment (Dapagliflozin alone vs. Dapagliflozin and Saxagliptin Combination vs. placebo), and time (v1, v2, v3), and the treatment by time interaction. This method allows us to use all non-missing subject data and adjusts for within-subject auto-correlation. For variables with significant effects in the mixed models, we examined the means graphically. SAS (version 9.4, Cary, NC) was used for data analysis with *p* < 0.05 considered significant.

Since subjects were randomized to treatment, chance of baseline subject characterizations acting as confounders are minimized. Therefore, randomized control trials do not usually adjust for baseline differences. However, in small studies, imbalances may exist as a result of random group assignment and may thus function as mediators of an association between treatment and outcome. In this situation, one would need to adjust for the mediators in order to obtain the direct effect of treatment on outcome, which is the effect of interest.

Due to the nature of this pilot study, detailed statistical analysis was avoided.

## Results

### Primary outcome

The study population was representative of subjects with uncontrolled type 2 diabetes, but with no preexisting macro-vascular complications. All adverse effects that occurred throughout the duration of the study were either not related to the study medication and design or fell within the expected side effects profile for Dapagliflozin and Saxagliptin. Table 1 shows blood biochemistry measures across the three visits. No statistical significance was observed between the groups for body composition measures. No significant difference in Body fat either in Dapagliflozin and combo groups throughout the study (Clinical Parameter Table 1). This shows that the changes that we observed in cellular and serum-based outcome measures were independent of body composition changes.

### Venous blood biochemistries

Venous blood biochemistries were gathered, both through Labcorp of America and through serum ELISA and both standard of care and research labs were collected.

**Fig. 2:**
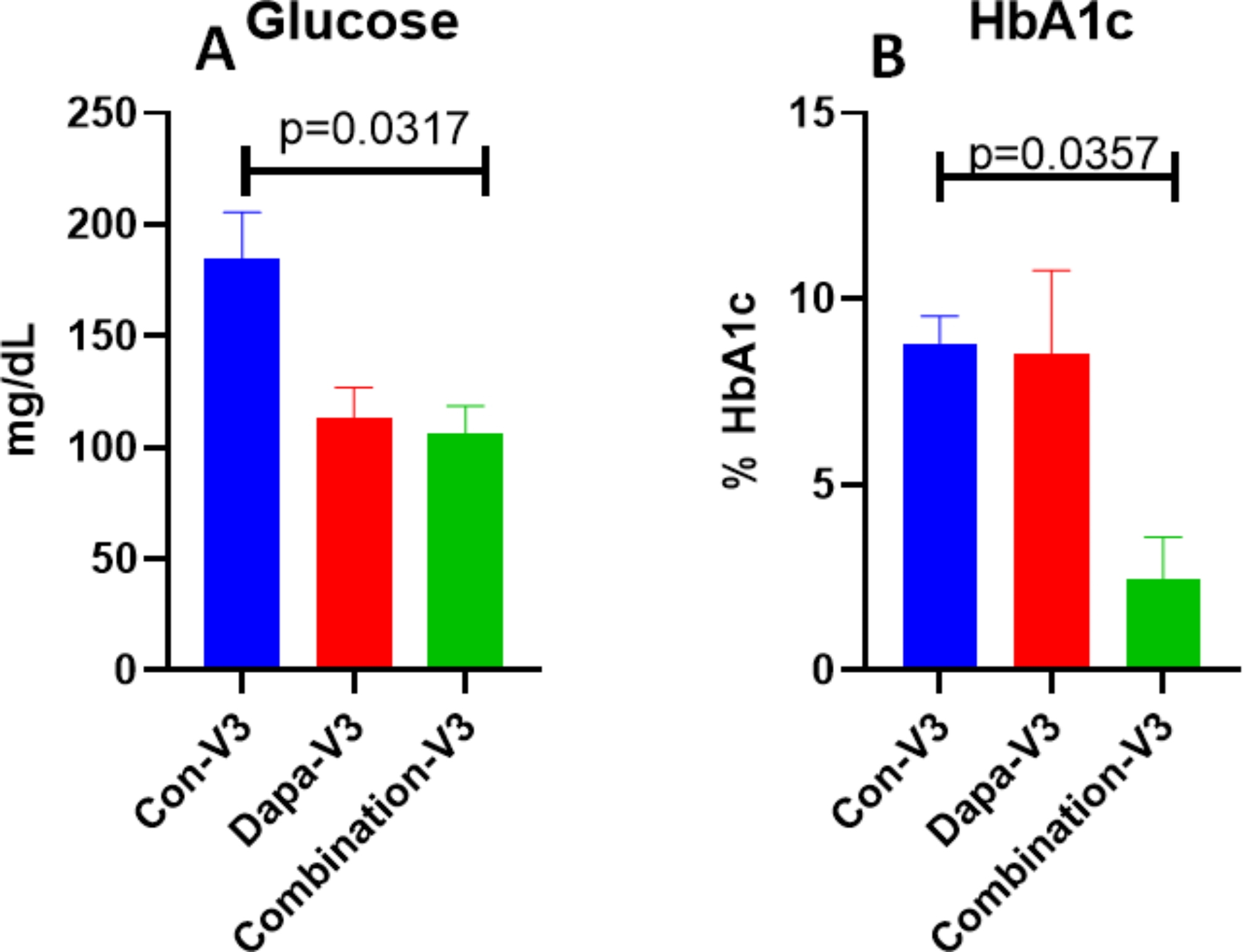
Both glucose and HbA1C levels decreased significantly in Combo Visit-3 as compared to Con-V3. The decreased glucose values in Dapa Visit-3 is not significantly different compared to Combo visit 3

**Fig. 3:**
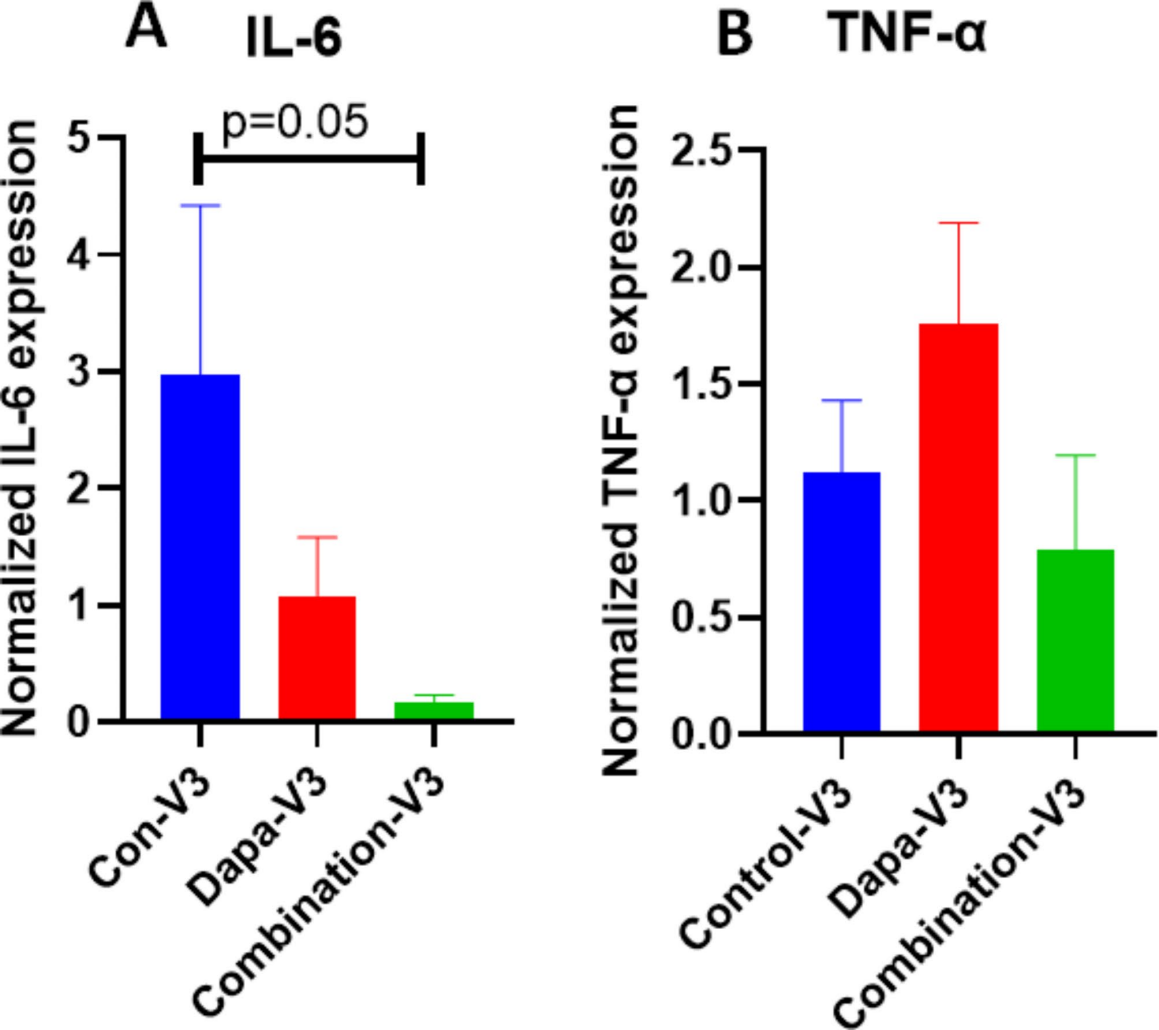
Gene expression of inflammatory markers- IL6 and TNFalpha. **A**) Gene expression levels of Inflammatory marker IL-6 decreased significantly in Combo-Visit-3 as compared to Control-visit-3. (**B**) No significant change in TNF-α expression between the groups

**Fig. 4:**
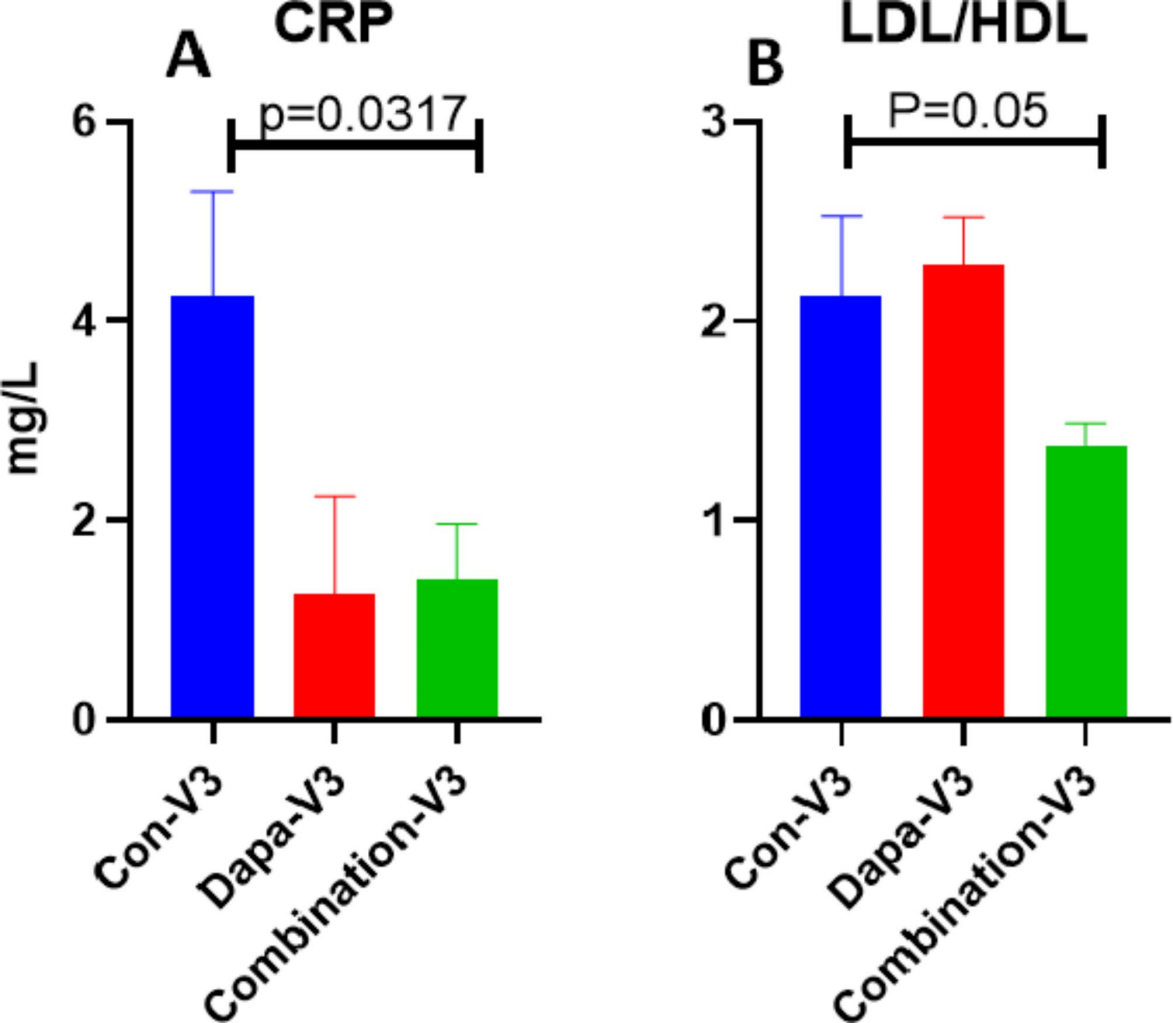
C-reactive protein (CRP) levels decreased in both the groups but is significantly decreased in Combo group- visit3 as compared to Control-group-visit-3. LDL/HDL levels decreased significantly on combo group-V3 as compared to Control group- Visit3

Detailed lab values of selected significant parameter are on Table 1. We found statistically significant decrease in HbA1c levels on visit-3 were observed in combo group(*p* = 0.03) as compared to Dapagliflozin alone and placebo group (Fig. 2) as compared to visit 1 and 2. The inflammatory marker IL-6 was significantly reduced in combo group visit-3(*p* = 0.05), whereas no significant change in TNF-α expression was observed (Fig. 3). Another inflammatory C-reactive protein (CRP) levels in serum also reduced significantly (Fig. 4A) on visit-3 as compared to visit 1 and 2 in Dapagliflozin alone and combo groups as compared to placebo group. Interestingly, we observed a significant reduction in lipid parameters, particularly triglyceride (TG) in Dapagliflozin alone group when compared to placebo group at visit 3 (visit 3 values at 99.5 *±* 7.2 vs. 129 *±* 12.3) and the ratio of LDL/HDL was also similar at 2.1 between these groups ( visit 3 values at 8 *±* 0.08 vs. 2.13 *±* 0.15). The LDL/HDL ratio is significantly reduced (*p* = 0.05) in combination group as compared to either Dapagliflozin alone or Placebo group (Fig. 4B).

**Fig. 5:**
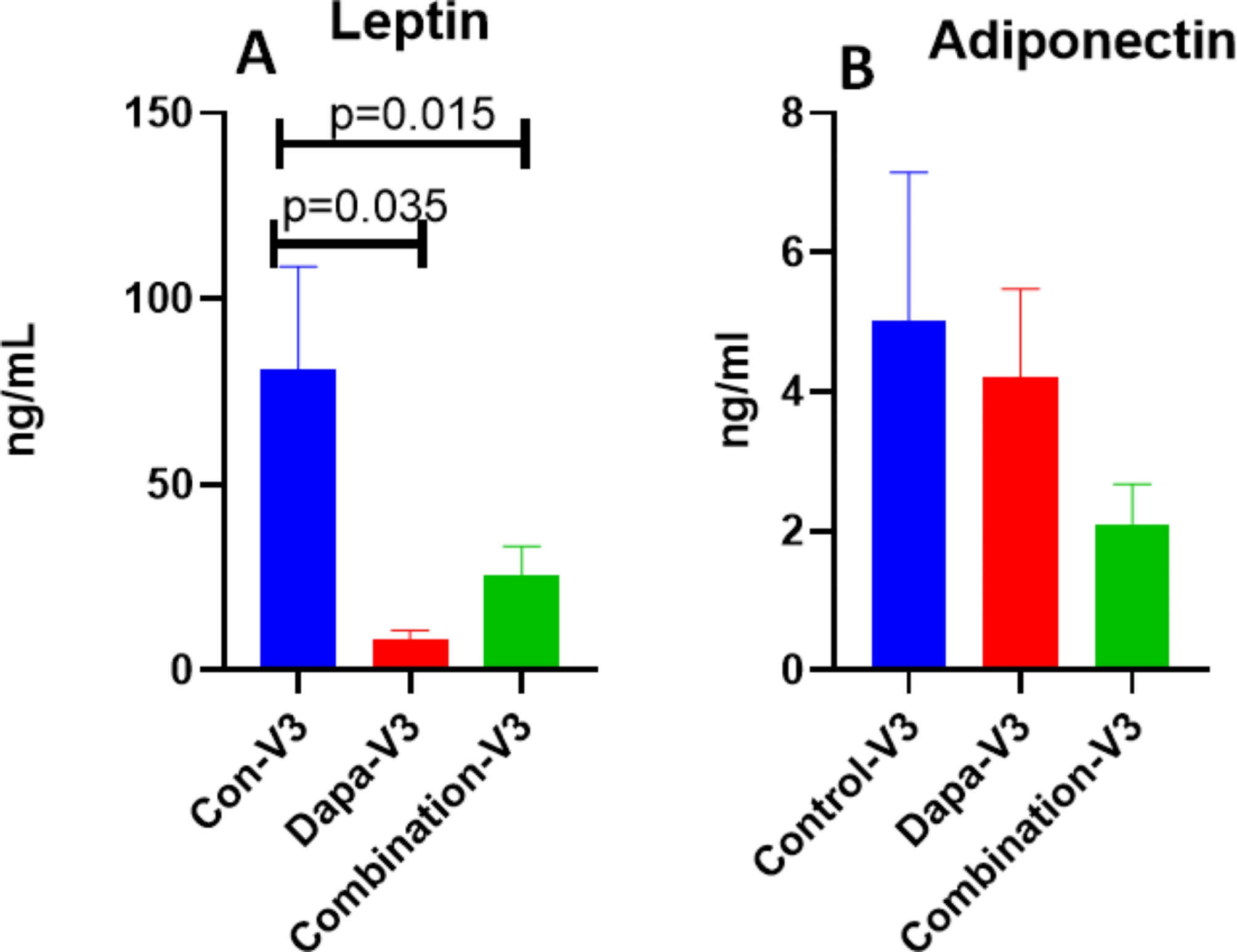
(**A**) Serum Leptin levels decreased significantly in both Dapa –Visit3 and Combo -Visit3 as compared to Control group Visit3. (**B**) No significant difference in adiponectin levels between the groups, though combination appears to have the lowest values

As shown in Fig. 5A, a significant reduction in Leptin levels were observed in both Dapagliflozin alone(*p* = 0.03) and Combo(*p* = 0.01) groups as compared to placebo group at visit-3 in comparison with visit 1 and 2. Whereas, adiponectin levels were higher in Dapagliflozin alone group but not showing any significant difference between the 3 groups (see Fig. 5B).

### Arterial stiffness

Stiffness of an artery is associated with cardiovascular diseases in older individuals and is positively associated with hypertension, coronary artery disease, stroke, heart failure and atrial fibrillation [20]. Arterial stiffness is assessed using standardized parameters such as Arterial stiffness adjusted for a heart rate of 75 (AI-75) and PWV. A trend in increased mean PWV was observed in combo group from visit-1 to visit-3 but was not statistically significant. Whereas no difference was observed in the other two groups between the visits.

### Adiposity

Body composition measurement showed no statistically significant change amongst the group throughout the visits. As expected, given short duration of the treatment the subjects were asked to maintain activity level as advised by American Diabetic Association (ADA) for healthy living [21]. There was no statistically significant change in hip to waist ratio, body weight and body fat percentage amongst both Dapa alone and Combo groups compared to placebo group.

### Outcome measures

#### Cellular Outcome measures

##### Count of CD34 + progenitor cells

To find out the effect of Dapagliflozin group and Combo group on the endothelial progenitor cell number we counted the total CD34 + ve cells by using Cellometer Mini (Nexcelom Biosciences, Lawrence, MA), after microbead column separation (BD Biosciences, San Jose, CA). As shown in Table 2, there is no statistical significance in difference in CD34 + ve cell numbers between Dapagliflozin, Combo and placebo groups.

##### Migration response

**Fig. 6:**
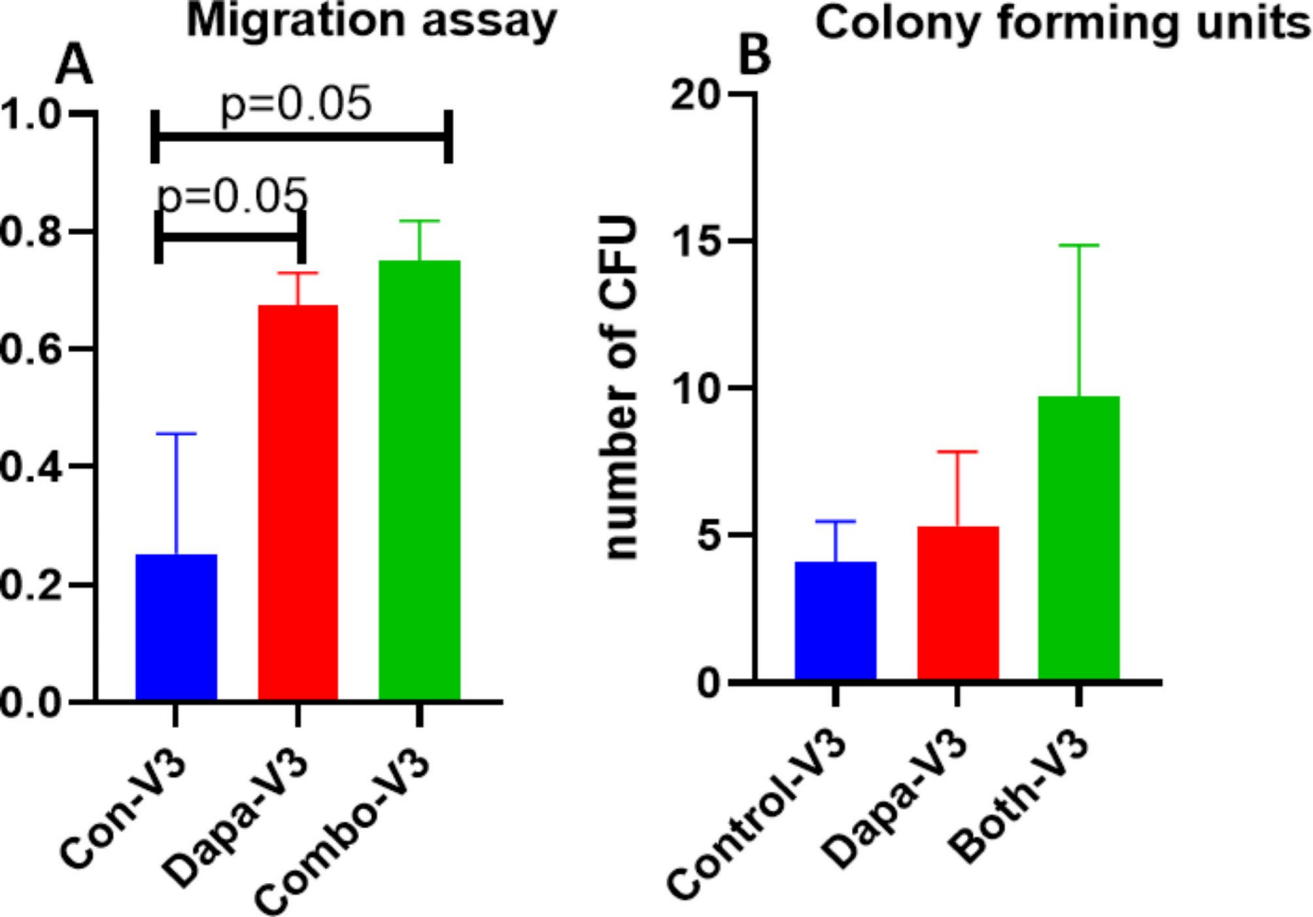
Migration Assay in response to SDF1α at 10ng/ml concentration and gene expression of SDF1a receptor CXCR-4 on CD34 + ve cells: (**A**) Mean migration of CD34 + ve cells towards SDF1- α significantly increased from Con-visit-3 to Dapa-Visit-3 and Con-visit-3 to Combo-Visit-3. That is there is no difference Dapa and Combo for migration assay (**B**) No significant change in colony forming units between the groups

The migratory response of CD34 + ve cells to the chemotactic factor SDF1α (concentration of 10 ng/ml ) increased significantly in both Dapa (*p* = 0.05)alone and Combo (*p* = 0.05) groups compared to placebo group(Fig. 6A).

##### CFU‑Hill’s colonies

No significant difference in CFU-Hill colony formation was observed between the groups was observed (Fig. 6B). There is a trend in increase the number of colonies was observed in combo group from Visit 1 to visit 2 and further decrease in colonies at visit 3 was observed.

### Gene expression analysis

#### The effect of dapagliflozin and combo group on the gene expression of CD34 + ve cells

We wanted to study the gene expression on CD34 + ve cells for antioxidants SOD2 (superoxide dismutase 2), GPX3 (glutathione peroxidase 3), CAT (catalase). The mean fold change in gene expression of all three antioxidants were not significant in both Dapagliflozin and Combo groups as compared to placebo group. Again, no significant difference between the mean gene expression for endothelial markers VEGF-A, KDR and PECAM1 in both Dapagliflozin and Combo groups as compared to placebo group from visit 1 to visit 3. We also checked the inflammatory markers IL6 and TNF-α expression. We observed a trend in decrease in the expression levels of these inflammatory markers from visit-1 to visit 3 between the groups when compared to placebo group.

### Urine podocyte function marker

#### Quantification of exosomal proteins in urine samples by western blot

**Fig. 7:**
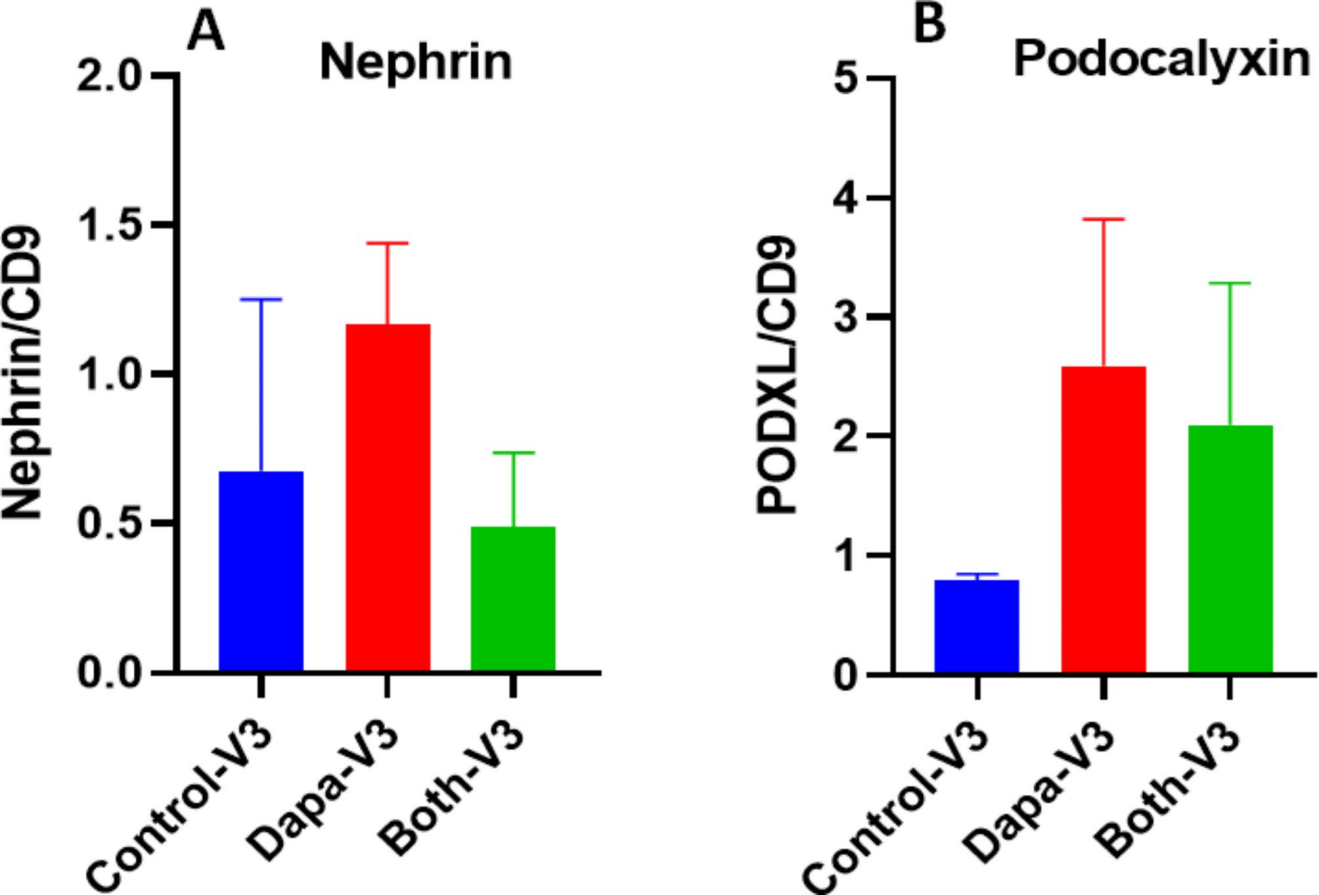
Urine exosomal analysis. The ratio of Nephrin/ CD9 (**A**) and Podocalyxin/CD9 (**B**) levels in urine was analyzed by western blot analysis. No significant difference between the groups was observed with either podocyte specific proteins

In our previous studies using Linagliptin and Canagliflozin [9, 10] we demonstrated that urine exosome-based podocyte specific proteins can serve as a useful renal function biomarker which is more sensitive than measuring proteinuria/albuminuria. Higher levels of podocyte inflammation is associated with increased podocytopathy and vice versa. We were interested to study the effect of Dapagliflozin and Dapagliflozin-Saxagliptin combination groups on podocyte health by quantifying the exosome expression for Nephrin, Renal Wilm’s Tumor (WT-1) and Podocalyxin like protein 1( PODXL) in urine samples. As described in the methods after isolating exosomes from urine samples from Dapagliflozin, Dapagliflozin-Saxagliptin and placebo groups we estimated amounts of proteins in the exosomes by western blot. Exosomes were identified by western blot with presence of CD9 positive where CD9 levels were used as a denominator. We noted lower levels of Nephrin in Dapagliflozin group though it did not reach statistical difference (see Table 2, Nephrin). The other proteins did not show any significant difference in band intensities between the groups for the podocyte proteins such as Podocalyxin from visit 1 to 3. The protein estimation of Podocalyxin and Nephrin are shown in Fig. 7.

Details of all cellular outcome measures are shown in Table 2. We did not notice any significant differences in UACR (urine albumin to creatinine ratio) between the groups.

### Characterization of endothelial progenitor cells (CD34 + ve) by flow cytometry

**Fig. 8:**
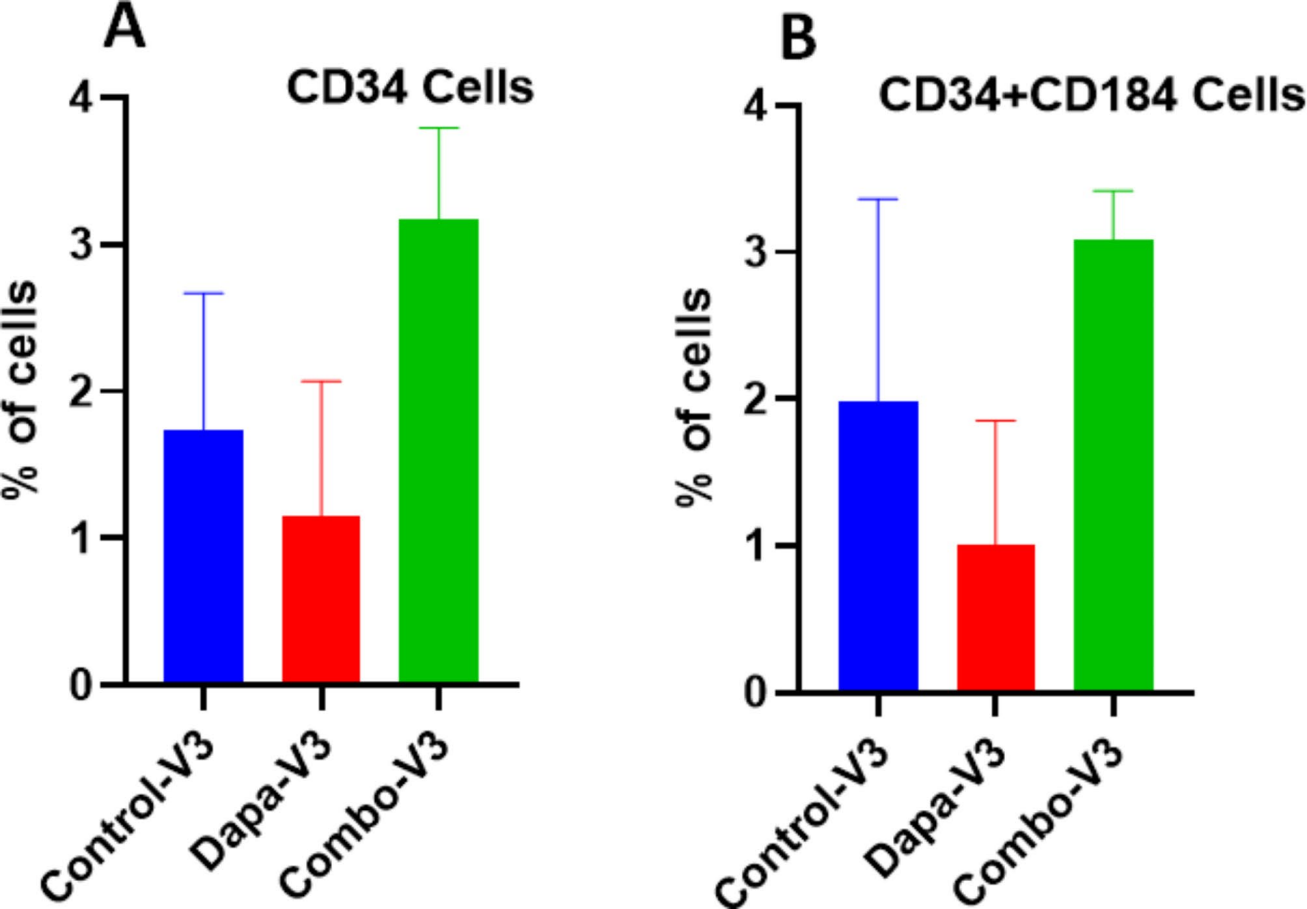
Flow cytometric analysis. The progenitor cells were analyzed by FACS. There was no significant difference in cell number (CD34 cells (**A**) and CD34+ CD184 cells) between the groups was observed. No difference between Dapa and Combo groups

Lastly Flow cytometric analysis of MNC was done. Progenitor cell count of CD34 + ve cells and CD34 + ve and CD184 dual positive cell count by flow cytometry is shown in Fig. 8. An increase in CD34 + ve and CD34/CD184 + + ve cells were observed from visit-1 to visit-3 in both Dapagliflozin and Combo groups as compared to placebo group, however that difference did not meet statistical difference. CD34 + is a progenitor cell marker and CD184 identifies the receptor for SDF1a chemotactic factor. The dual positive cells identify a sub-population of MNC that are CD34 + ve and also will be able to respond to a chemotactic factor such as SDF1a, indicating a progenitor cell population that are able to migrate and thereby able to reach a site that needs vascular repair.

Incidentally CD31 ( a mature endothelial cell marker) flow cytometric analysis showed a similar trend as CD34 + across the groups (see Table 2).

## Discussion

In this small pilot study of 15 subjects, we evaluated the combined effect of dapagliflozin with saxagliptin to study changes on endothelial function in Type 2 DM patients. We considered three regimens: dapagliflozin alone or in combination with saxagliptin and placebo group.

We have observed that SGLT-2 inhibitor- Dapagliflozin, plus the DPP‐4 inhibitor -Saxagliptin combination group led to greater improvements in glycemic control than add‐on agent Dapagliflozin alone. These findings are in accordance with previous studies [11–13]. Similarly inflammatory markers were also lower in the combination arm.

Interestingly the endothelial progenitor cell number did not change significantly but has shown a non-significant increase in number in Dapagliflozin alone group. In this group the migration function improved significantly in Dapagliflozin alone as well, and also in combination groups compared to control group. Several reports have shown the efficacy of the combination of SGLT2 and DPP-4 inhibitors in patients with type 2 diabetes [11, 22]. Interestingly LDL/HDL ratio was also better in combination. However, the potential of this combination to improve vascular function beyond glycemic control has not been explored until now.

Several parameters showed significant improvement with *both* Dapagliflozin alone and Combination compared to placebo group such as HbA1C, fasting glucose, CRP, IL6, and Leptin reduction. There was also improvement in CD34 + number, EPC migration and colony formation unit counts.

Therefore, other than glycemic control, the combination did offer benefit over Dapagliflozin alone in inflammatory markers and LDL/HDL ratio, however the cellular parameters (CD34 + number and migration) showed similar results between Dapa and combination compared to placebo as far as all cardio-metabolic parameters taken together.

We noted slightly lower levels of Nephrin protein in urine exosome samples from Dapa group alone compared to Dapa + Saxa combination group, though it did not reach statistical significant difference. However, this finding raises question whether the combination fails to protect the kidneys when compared to Dapagliflozin alone. No similar trend was noted with podocalyxin.

Though this is a small study with 15 patients with approximately 5 subjects in each group.

### Summary

This is a pilot study which is based on 4–5 subjects in each of the three arms. We have looked at more than 20 different parameters between cellular and non-cellular outcome measures of which several parameters showed statistically significant differences compared to placebo, for example migration assay, LDL/HDL ratio, serum Leptin, hs-CRP, Interleukin-6 and nearly significance with urine podocalyxin value (a marker for podocyte inflammation). These results indicate that there is an effect on cardio-kidney-metabolic (CKM) effect of dapagliflozin and Dapa-Saxa combination compared to placebo. Moreover, there is a statistically significant difference in both glycemic indices of HbA1C and fasting glucose, favoring the combination, which clinically is a relevant finding. The fact that there was no change in body fat by Tanita scale and weight indicates that these medication effects on cellular and serum parameters are not always reflected in change in body composition indices [21].

This study is important for clinical guidance in diabetes practice regarding use of monotherapy versus combination therapy particularly to address cardiometabolic benefit with novel diabetes medications. Though the present study has a relatively small sample size and future studies are warranted to compare their effects in a larger prospective trial, the study highlights that use combination of SGLT2i and DPP4i in clinical practice maybe beneficial as far as glycemic control and lipid parameters are concerned. However these benefits did not pass on to cellular outcome measures in T2DM with CKD patients.

## Conclusion

Contrary to our initial hypothesis, the combination does not offer any benefit towards cellular parameters of cardiovascular risk beyond singular agent of SGLT2-inhibitors. It is therefore recommended to use Dapagliflozin alone rather than in combination with DPP4 inhibitor like saxagliptin in clinical care of T2DM with CKD patients to address all aspects of CKM while keeping the therapeutic cost lower. Long term studies with parameters for heart failure should be helpful in this context. It is however important to emphasize that Dapagliflozin alone did show improvement in all parameters of cardiovascular outcome measures compared to placebo and should be concerned in high risk T2DM patients with complications like CKD.

## Data Availability

All relevant data generated or analyzed during this study are included in this published article in the form of figures, legends and discussion. All relevant data has been disclosed in Tables 1 and 2 as clinical and cellular data. Any other relevant will be available on request. For all human participants (including the use of tissue samples), informed consent has been obtained from all subjects and/or their legal guardian(s).
